# Transitions of care interventions to improve quality of life among patients hospitalized with acute conditions: a systematic literature review

**DOI:** 10.1186/s12955-021-01672-5

**Published:** 2021-01-29

**Authors:** Tolu O. Oyesanya, Callan Loflin, Lindsey Byom, Gabrielle Harris, Kaitlyn Daly, Lesley Rink, Janet Prvu Bettger

**Affiliations:** 1 Duke University School of Nursing, 307 Trent Dr., Durham, NC 27710 USA; 2 Duke University School of Medicine, Durham, NC USA; 3 Department of Allied Health Sciences, University of North Carolina-Chapel Hill, Chapel Hill, NC USA

**Keywords:** Transitional care, Patient care, Quality of life

## Abstract

**Background:**

Although transitional care interventions can improve health among patients hospitalized with acute conditions, few interventions use patient quality of life (QOL) as the primary outcome. Existing interventions use a variety of intervention components, are not effective for patients of all races and ethnicities, do not address age-related patient needs, and do not incorporate the needs of families. The purpose of this study was to systematically review characteristics of transitional care intervention studies that aimed to improve QOL for younger adult patients of all race and ethnicities who were hospitalized with acute conditions.

**Methods:**

A systematic review was conducted of empirical literature available in PubMed, Embase, CINAHL, and PsycINFO by November 19, 2019 to identify studies of hospital to home care transitions with QOL as the primary outcome. Data extraction on study design and intervention components was limited to studies of patients aged 18–64.

**Results:**

Nineteen articles comprising 17 studies met inclusion criteria. There were a total of 3,122 patients across all studies (range: 28–536). Populations of focus included cardiovascular disease, chronic obstructive pulmonary disease, stroke, breast cancer, and kidney disease. Seven QOL instruments were identified. All interventions were multi-component with a total of 31 different strategies used. Most interventions were facilitated by a registered nurse. Seven studies discussed intervention facilitator training and eight discussed intervention materials utilized. No studies specified cultural tailoring of interventions or analyzed findings by racial/ethnic subgroup.

**Conclusions:**

Future research is needed to determine which intervention components, either in isolation or in combination, are effective in improving QOL. Future studies should also elaborate on the background and training of intervention facilitators and on materials utilized and may also consider incorporating differences in culture, race and ethnicity into all phases of the research process in an effort to address and reduce any health disparities.

## Introduction

Transitional care is defined as actions in the clinical encounter designed to ensure the coordination and continuity of healthcare for patients transferring between different locations or levels of care [1]. There are many factors that contribute to gaps in the transition from hospital to home, including inadequate planning, insufficient patient/family education, and limited and fragmented access to essential services [2]. These gaps are often compounded by limited financial resources, inadequate insurance coverage [3], and lack of social support [4].

Acute conditions are sudden, severe in their onset, and often require immediate medical attention that results in hospital admission [5]. Patients who are hospitalized for acute conditions and their families could benefit from transitional care [6, 7], as transitional care management in a variety of patient populations has led to improved patient and family outcomes, including improved patient QOL [3, 8]. However, there are limited standards to guide transitional care of patients with acute conditions and the current state of “usual care” during the transition from acute hospital care to home has limited provider support or engagement to help patients and families navigate and access fragmented health and community-based services during the transition home from the hospital [9, 10]. Of the existing transitional care intervention for patients hospitalized for acute conditions, few focus on patient QOL as the primary outcome. In addition, existing interventions use a variety of different intervention components, are not effective for patients of all races and ethnicities, do not address age-related differences in patient needs, and do incorporate family needs [11–15].

To address these gaps in the literature, we draw from literature on transitional care interventions designed to improve QOL for younger adult patients (aged 18–64) hospitalized with acute conditions to inform development of transitional care interventions to improve QOL in patients with acute conditions. We emulate research on transitional care interventions for older adult patients with chronic conditions [16, 17] by focusing solely on transitional care interventions for younger adult patients hospitalized for acute conditions. Thus, the purpose of this systematic literature review was to determine characteristics of transitional care intervention studies designed to improve QOL for younger adult patients (age 18–64) of different racial and ethnic groups who were hospitalized for an acute condition and discharged home.

## Methods

The design and dissemination of findings for this systematic literature review followed the Preferred Reporting Items for Systemic Review and Meta-Analysis (PRISMA) statement [18]. The study protocol was documented in the International Prospective Register of Systematic Reviews (PROSPERO), Registration Number: CRD42020147345 [19].

### Information sources

Search terms for the study were initially developed with the study team in consultation with a medical librarian who specializes in systematic literature reviews. Pre-defined search terms were used to identify articles describing transitional care interventions used to improve QOL in younger adult patients with acute conditions or injuries. These included specific terms related to transitional care, hospitalization, outpatient care, and quality of life. The search strategy was reviewed by a second medical librarian before searching the following databases: PubMed, Embase, The Cumulative Index to Nursing and Allied Health Literature (CINHAL), and PsycINFO. The final search was conducted on November 19, 2019. The search excluded studies not published in English and studies of animals; we also excluded editorials, letters, case reports, and commentaries. The full electronic search strategy for one database (PubMed) is contained in Additional file 1.

### Study eligibility characteristics

Inclusion criteria for studies included in this review:

1. Patients in the sample were:
  - age 18–64 years [as the age-related needs and outcomes of younger and older adults differ [20, 21]]
  - hospitalized in the last 30 days due to acute condition or injury
  - discharged to home/community from the acute hospital (intensive care unit, acute, or subacute unit) or a subsequent inpatient location (such as inpatient rehabilitation)
2. Family members participating in the study (if applicable) were:
  -  ≥ 18 years
  - the anticipated, informal primary caregiver in unpaid role for a patient who meets eligibility based on patient criteria above
  - informal primary caregivers included immediate or extended family members or close friends of patient
3. Primary study outcome: patient QOL

Exclusion criteria for studies:

1. Patients in the sample:
  - had a planned hospitalization
  - were exclusively ≤ 17 and ≥ 65 years
2. Family members participating in the study (if applicable):
  - had no plans to live with patient or spend ≥ 10 h per week with patient
  - were in a paid caregiving position
3. QOL was not explicitly mentioned as the sole primary outcome (unless article was needed to provide context to a study that met inclusion criteria)

### Study selection

Studies identified in each database were imported into Covidence [22], a systematic review software, to facilitate study selection. Duplicates were removed. Five researchers independently screened titles and abstracts against the inclusion and exclusion criteria. Two authors evaluated each title/abstract, and there was 98% complete agreement between reviewers. A third reviewer who was not part of the title/abstract review resolved conflicts. The articles that advanced to full-text review were evaluated by two reviewers to select which studies met eligibility for data extraction and quality assessment.

### Data collection process

The team collaborated to determine the data of interest from all studies prior to full-text article review. A draft data extraction spreadsheet was piloted with two full-text articles with the full team before finalizing the list of data to extract. We used a three-tiered review to extract data from the full-text articles. In the first-level, two researchers led a structural review, independently extracting the following information into a spreadsheet from each full-text article, including the article citation, country of study, study design, health condition of focus, inclusion/exclusion criteria, treatment intervention description, and QOL outcome measurement (i.e., name of measure). The second level of review used a content analysis to fully describe the treatment intervention description from each article specifying individual intervention components, facilitator type and training, intervention materials, and cultural tailoring. In the final-level of review, four researchers extracted participant characteristics and QOL-specific findings, including whether main findings were described by racial/ethnic subgroup. A single researcher extracted data from each assigned article and a second researcher audited the data extracted. Consensus was reached through discussion.

### Quality assessment

The QualSyst Tool was used to evaluate methodological quality of each study that met the criteria for study inclusion [23]. The QualSyst Tool includes 14 criteria for quantitative studies, with each criterion being rated yes (2 points), partial (1 point), no (0 points), or not applicable [23]. Based on the QualSyst Tool’s criteria, each study is given a summary score, which is equivalent to the total sum divided by the total possible sum. The highest possible score for each study is 1.0 [23]. The minimum threshold for summary scores is to be set by the research team and should be based on the distribution of summary scores for articles included in the review [23]. We set our minimum threshold at 0.70. Quality assessment was conducted in pairs, whereby a single researcher assessed the quality of assigned articles and a second researcher audited the quality assessment. Disagreements in quality assessment were discussed until consensus was reached.

## Results

### Study selection

**Fig. 1:**
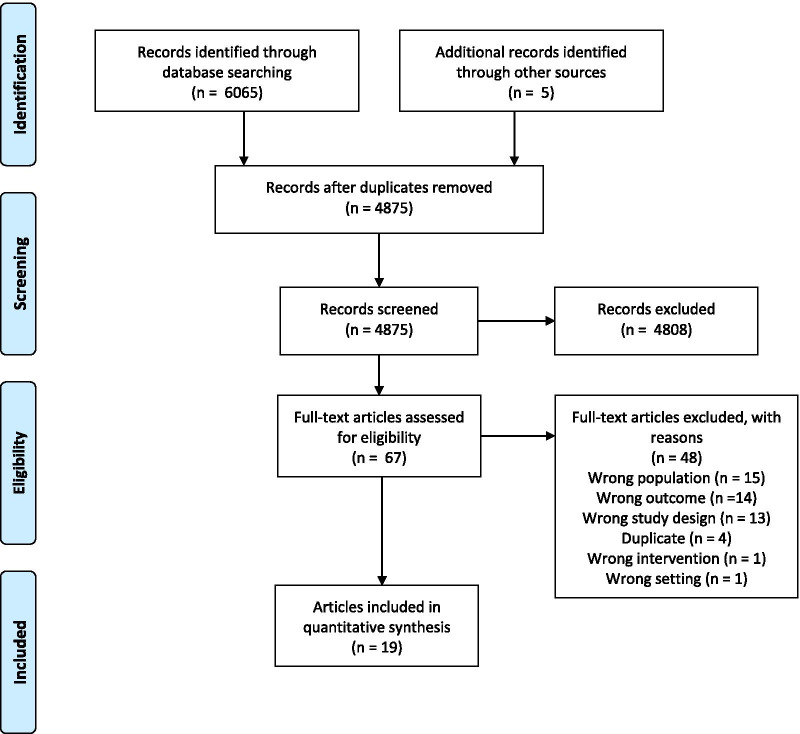
Flow diagram

The literature search yielded 6,070 titles and abstracts, and of those, 1,195 were duplicates. Of 4,875 unique titles and abstracts, 4,808 did not meet inclusion criteria, resulting in 67 articles for full-text screening. After determining the eligibility of the full-text articles, we included 19 for data extraction and quality assessment (see Fig. 1, PRISMA Flow Diagram). Excluded full-text articles are listed in Additional file 1. Two papers (Aboumatar et al. [24] and Weintraub et al. [25]) were included to provide additional context about the intervention description) that was not described in associated study papers (Aboumatar et al. [26] and Konstam et al. [27], respectively). Therefore, our review contains 17 unique studies.

### Study characteristics

The 17 studies reviewed represent 10 countries (Table 1). There were four from the United States [24–29]; two each from Norway [30, 31], China [32, 33], Canada [34, 35], and Iran [36, 37]; and one each from Spain [38], Netherlands [39], Australia [40], Poland [41], and the United Kingdom [42]. Seven studies addressed patients with cardiovascular disease [27, 29, 31, 34–37, 41], four focused on stroke patients [28, 30, 33, 39], three studied patients with chronic obstructive pulmonary disease [26, 37, 40], and one each for patients with breast cancer [42] or with kidney disease [32]. All studies were randomized controlled trials [24–28, 30–37, 39–42] except one that used a quasi-experimental [38] and one that had prospective one group pre- and post-test design [29]. QOL was measured using seven different measures, including the Saint George's Respiratory Questionnaire (SGRQ) [26, 38, 40], Short-Form 36 (SF-36) [27, 28, 31, 33, 35, 39], Minnesota Living with Heart Failure Questionnaire (MLHFQ) [27, 29, 34, 37, 41], Nottingham Health Profile (NHP) [30], Kidney Disease Quality of Life Short Form (KDQOL-SFTM) [32], Functional Assessment of Cancer Therapy-Breast (FACT-B) [42], and EuroQOL-5D (EQ-5D) [42]. Quality assessment summary scores ranged from 0.714 to 0.964, which met our minimum threshold of 0.70 for summary scores. The range of scores implies most studies included in this review were middle to high quality. No studies were excluded based on the quality assessment summary score threshold.

**Table 1:** Study characteristics (N = 17 studies)

References	Country	Population of focus	Study design	Quality of Life Measure	Quality assessment summary score/ out of 1.0
Abad-Corpa et al. [38]	Spain	Chronic Obstructive Pulmonary Disease	Quasi-Experimental	Saint George's Respiratory Questionnaire (SGRQ)	.9090
Aboumatar et al. [24]^a^ and [26]	United States	Chronic Obstructive Pulmonary Disease	RCT	Saint George's Respiratory Questionnaire (SGRQ)	.9090; .9285
Boter [39]	Netherlands	Stroke	RCT	Short-Form-36 (SF-36)	.9285
Chow et al. [32]	China	Peritoneal dialysis/hemodialysis	RCT	Kidney Disease Quality of Life Short Form (KDQOL-SFTM)	.9642
Claiborne [28]	United States	Stroke	RCT	Short-Form-36 (SF-36)	.75
Fjærtoft et al. [30]	Norway	Stroke	RCT	Nottingham Health Profile (NHP)	.857
Hanssen et al. [31]	Norway	Myocardial infarction	RCT	Short-Form-36 (SF-36)	.821
Harrison et al. [34]	Canada	Congestive heart failure	RCT	Minnesota Living with Heart Failure Questionnaire (MLHFQ)	.964
Hermiz et al. [40]	Australia	Chronic Obstructive Pulmonary Disease	RCT	Saint George's Respiratory Questionnaire (SGRQ)	.785
Konstam et al. [27] and Weintraub et al. [25]^a^	United States	Congestive heart failure	RCT	Minnesota Living with Heart Failure Questionnaire (MLHFQ)	.785;.892
Mehralian et al. [36]	Iran	Congestive heart failure	RCT	Short-Form-36 (SF-36)	.821
Rezapour-Nasrabad [37]	Iran	Chronic heart failure	RCT	Minnesota Living with Heart Failure Questionnaire (MLHFQ)	.785
Wells et al. [42]	United Kingdom	Breast cancer	RCT	Functional Assessment of Cancer Therapy-Breast (FACT-B) and EQ-5D	.884
Whitaker-Brown et al. [29]	United States	Heart failure	Prospective one group pre- and post-test design	Minnesota Living with Heart Failure Questionnaire (MLHFQ)	.95
Wierzchowiecki et al. [41]	Poland	Chronic heart failure	RCT	Minnesota Living with Heart Failure Questionnaire (MLHFQ)	.714
Wong et al. [33]	China	Stroke	RCT	Short-Form-36 (SF-36)	.892
Woodend et al. [35]	Canada	Cardiac disease	RCT	Short-Form-36 (SF-36)	.821

RCT, randomized controlled trial

^a^Article included to provide context for related article

### Sample characteristics

Sample size across studies varied from 28 to 536 total participants (Table 2). Although the review focused on adults age 18–64, most samples had a mix of younger and older adults, with the mean age of treatment group participants ranging from 54.5 to 75.52 years (median across studies = 66.65). Six of the studies had samples where females composed ≥ 50% in the treatment group [26, 28, 29, 33, 39, 40]. The four studies from the United States reported the race and ethnicity of study participants by study arm [25–29], but no studies reported main findings by race or ethnic group. Nine studies reported the percentage of participants who were married by study arm [28, 29, 31–34, 36, 37, 42]. Ten studies reported the education level or proxy of education, including varying categories of educational levels or mean number of years of education [26, 28, 31–34, 37–39, 42]. Four studies reported participant income levels or a proxy of income, including reporting participants’ mean annual household income, varying levels of income categories, and participants’ perceptions of adequacy of their financial status [26, 28, 32, 38]. Only one study reported the health insurance status of participants [28], and nine studies were conducted in countries with universal health care [30, 31, 34, 35, 38–42].

**Table 2:** Sample characteristics

References	Number of participants	Age by study arm [Mean (SD)]	Sex by study arm [N (%) female]	Race/ethnicity by study arm [N (%)]	Marital Status by study arm [N (%) married]	Report of education levels	Report of income levels	Report of health insurance status	Sub-group analysis of results by race/ethnicity
Abad-Corpa et al. [38]	Total: 143 Treatment: 56 Control: 87	Treatment: 71.61 (8.37) Control: 73.51 (7.77)	Treatment: 6 (10.7%) Control: 7 (8%)	NR	DS	R	PR	NR	NR
Aboumatar et al. [26]	Total: 240 Treatment: 120 Control: 120	Treatment: 63.9 (9.6) Control: 66 (10)	Treatment: 72 (60%) Control: 76 (63.3%)	Treatment: - White: 98 (81.6%) - African American: 20 (16.7%) - American Indian/Alaska Native: 2 (1.7%) Control: - White: 100 (83.3%) - African American: 18 (15%) - American Indian/Alaska Native: 2 (1.7%)	NR	R	R	NR	NR
Boter et al. [39]	Total: 536 Treatment: 263 Control: 273	DS	Treatment: 133 (51%) Control: 143 (52%)	NR	NR	R	NR	NR	NR
Chow et al. [32]	Total: 85 Treatment: 43 Control: 42	Treatment: 54.5 (12.8) Control: 59.4 (13.97)	Treatment: 18 (42.9%) Control: 15 (34.9%)	NR	Treatment: 28 (65.1%) Control: 33 (78.6%)	R	PR	NR	NR
Claiborne [28]	Total: 28 Treatment: 16 Control: 12	Treatment: 70 (13.97) Control: 65 (11.99)	Treatment: 9 (56.25%) Control: 31 (23.5%)	Treatment: - White: 12 (75%) - African American: 4 (25%) Control: - White: 12 (100%)	Treatment: 13 (81.25%) Control: 9 (75%)	R	R	R	NR
Fjærtoft et al. [30]	Total: 320 Treatment: 160 Control: 160	NR	NR	NR	NR	NR	NR	NR	NR
Hanssen et al. [31]	Total: 288 Treatment: 156 Control: 132	Treatment: 59.5 (12.9) Control: 60.9 (10.8)	Treatment: 24 (15.4%) Control: 31 (23.5%)	NR	Treatment: 118 (75.6%) Control: 107 (82.3%)	R	NR	NR	NR
Harrison et al. [34]	Total: 192 Treatment: 92 Control: 100	Treatment: 75.52 (10.41) Control: 75.74 (9.40)	Treatment: 43 (47%) Control: 44 (44%)	NR	Treatment: 38 (41%) Control: 43 (43%)	R	NR	NR	NR
Hermiz et al. [40]	Total: 177 Treatment: 84 Control: 93	DS	Treatment: 43 (51.2%) Control: 50 (53.8%)	NR	NR	NR	NR	NR	NR
Konstam et al. [27]	Total: 88 Treatment: 44 Control: 44	Treatment: 71.67 (11.98) Control: 66.93 (13.10)	Treatment: 18 (40.9%) Control: 14 (31.8%)	Treatment: - Black: 4 (9.3%) - Hispanic: 1 (2.3%) - White: 38 (88.4%) Control: - Black: 4 (9.1%) - Hispanic: 2 (4.5%) - Native American: 1 (2.3%) - White: 37 (84.1%)	NR	NR	NR	NR	NR
Mehralian et al. [36]	Total: 110 Treatment: 55 Control: 55	Treatment: 61.28 (13) Control: 62.7 (10)	DS	NR	Treatment: (100%)^a^ Control: (96%)^a^	NR	NR	NR	NR
Rezapour-Nasrabad [37]	Total: 168 Treatment: 83 Control: 85	DS	Treatment: 31 (37.3%) Control: 31 (36%)	NR	Treatment: 67 (89.3%) Control: 70 (95.9%)	R	NR	NR	NR
Wells et al. [42]	Total: 108 Treatment: 54 Control: 54	Treatment: 54.9 (12.23) Control: 57.3 (8.66)	DS	NR	Treatment: 40 (74.1%) Control: 38 (70.4%)	PR	NR	NR	NR
Whitaker-Brown et al. [29]	Total: 50 Treatment: 50 Control: 0	Treatment: 70.1 (11.7) No control	Treatment: 21 (58%) No control	Treatment: - Caucasian: 30 (83%) - African American: 6 (17%) No control	Treatment: 18 (50%) No control	NR	NR	NR	NR
Wierzchowiecki et al. [41]	Total: 160 Treatment: 80 Control: 80	Treatment: 67.3 (10.2) Control: 69.5 (10.7)	Treatment: 32 (40%) Control: 33 (41%)	NR	NR	NR	NR	NR	NR
Wong et al. [33]	Total: 108 Treatment: 54 Control: 54	Treatment: 67.5 (11.6) Control: 71.5 (11.6)	Treatment: 34 (63%) Control: 34 (63%)	NR	Treatment: 37 (68.5%) Control: 29 (53.7%)	PR	NR	NR	NR
Woodend et al. [35]	Total: 249 Treatment: 125 Control: 124	Heart Failure Group-Treatment 67 (13) Control: 66 (11) Angina Group-Treatment: 66 (12) Control: 65 (10)	DS	NR	NR	NR	NR	NR	NR

NR, not reported; DS, variable reported in different structure; R, reported; PR, proxy of variable reported

^a^N not reported

### Transitional care intervention characteristics

Transitional care intervention components and characteristics varied (Table 3). It was most common for interventions to be facilitated by a nurse (n = 13) [24–27, 31–40, 42], an interdisciplinary team (n = 3) [29, 30, 41], or a social worker (n = 1) [28]. Seven studies reported on the training of the intervention facilitator [24–26, 31–33, 36, 38, 39], with varying level of detail on report of training. Eight studies reported of the intervention materials used, including materials for study participants and for facilitators [24, 32–36, 39–41]. All interventions were multi-component. There were 31 distinctive intervention components used across the 17 studies. The most common intervention components were: patient education (n = 16); phone calls (n = 12); patient needs assessments (n = 11); home visits (n = 10); family education (n = 7); referral to other services (n = 7); contact to primary care provider or other community providers (n = 7); and on-call provider line (n = 6). The category “other” included directives to intervention facilitators on support of patient; when to contact the patient; technical care; and maintaining electronic health records; and specification of whose care the patient is under [27, 33, 35–37, 41, 42]. No studies described how they culturally-tailored their intervention to address the racial/ethnic needs and preferences of the population.

**Table 3:** Transitional care intervention characteristics (N = 17 studies)

Intervention characteristics	Study citations	Number of studies
Type of facilitator		
Nurse(s)	[26, 27, 31–40, 42]	13
Social worker(s)	[28]	1
Interdisciplinary team	[29, 30, 41]	3
Training of facilitator		
Reported	[24–26, 31–33, 36, 38, 39]	7
Not reported	[27–30, 34, 35, 37, 40–42]	10
Intervention materials		
Reported	[24, 32–36, 39–41]	8
Not reported	[25–31, 37, 38, 42]	9
Intervention components		
Patient needs assessment	[24, 25, 28, 29, 31–33, 35, 38–42]	11
Family needs assessment	[28, 39]	2
Identification/involvement of family caregiver	[32, 33, 38, 42]	4
Problem-solving	[24, 28, 31, 39]	3
Phone calls	[24–29, 31–34, 37, 39, 41, 42]	12
Hospital visits	[24–26, 37, 38]	3
Clinic visits	[25, 29, 30, 41]	3
Home visits	[24, 26, 28, 30, 33, 36, 38–42]	10
Telehealth (i.e., video conferencing)	[35]	1
Discharge planning/preparation	[24, 26, 32, 34]	3
Patient education	[24–26, 28–42]	16
Family education	[24, 26, 28, 31, 32, 37–39]	7
Use of teach-back method	[24]	1
Distribution of educational materials	[24, 34, 36, 39–41]	5
Care coordination/patient navigation	[28, 29, 33, 41]	4
Referral to other services	[24, 28, 29, 32, 37, 38, 40, 41]	7
Development of nursing report	[24, 38]	1
Development of individualized health plan	[24, 30–32, 34, 38, 40]	6
Contact with primary care providers or other community providers	[25, 27, 29, 34, 38, 40–42]	7
Encourage patient to contact PCP	[31, 39, 40]	3
Motivational interviewing	[24, 32]	1
On-call provider line	[24, 25, 29, 31, 32, 37, 41, 42]	6
Monitoring patient progress	[25, 27, 28, 33, 35, 40]	5
Interdisciplinary rehabilitation/physical training	[30, 33, 41]	3
Goal setting	[31, 33]	2
Regular data collection	[25, 27, 29, 35, 41]	4
Distribution of medical records to patient	[42]	1
Medication reconciliation	[29, 41]	2
Medication management	[25, 29, 41]	2
Interdisciplinary cooperation	[24, 25, 29, 30, 34, 41]	4
Other	[27, 33, 35–37, 41, 42]	7
Cultural tailoring of intervention		0

Patient education typically included education about self-management of condition and expectations of recovery; family education included the same information in addition to information about the family member’s role in supporting the patient. Phone calls were often from the intervention facilitator to the patient after discharge to discuss patient needs, coordinate care or study activities, or provide education. Patient needs assessments occurred either pre- or post-discharge and included use of pre-set surveys or measures (standardized or developed by the study team) to determine patient cognitive, physical, emotional, and social needs. The results on patient needs assessment were often used to inform tailoring of the intervention based on patient needs or referral to additional services; limited detail was provided on the patient needs assessments utilized. Home visits consisted of visits by the intervention facilitator or other assigned providers to assess the patient in their home environment and/or provide in-person education. The patient’s primary care provider or other community providers were contacted by the intervention facilitator to provide an update on the patient’s progress and needs; however, there was varying timeframes of when this contact occurred. Finally, studies utilizing an on-call provider line directed patients to call a set phone number if they had any questions or concerns about their health. The on-call provider was often the intervention facilitator or provider. The time frame of on-call provider availability varied, including 24/7, business hours (Monday-Friday, 9 AM-5 PM), or after business hours.

## Discussion

The primary purpose of this systematic literature review was to determine characteristics of transitional care intervention studies designed to improve QOL for younger adult patients (age 18–64) of different racial and ethnic groups who were hospitalized for an acute condition and discharged home. Findings suggest wide variation on QOL instruments, intervention components, and description of facilitator training and intervention materials utilized in transitional care interventions for younger adult patients hospitalized with acute conditions. In addition, no studies specified cultural tailoring of interventions or analyzed findings by racial/ethnic subgroup.

Research has shown that transitional care interventions can address gaps in care with a variety of strategies, including individualized transitional care plans, post-discharge care coordination, and community-based service referrals [16, 43–46]. Although diverse populations of younger adult patients hospitalized for acute conditions were included in this review, findings showed there are common components integrated into transitional care interventions for these patients. More specifically, the present study found that patient and family education, phone calls to patients, patient needs assessments, and home visits were most common components of transitional care interventions for younger adult patients with acute conditions. However, no studies specified which intervention component(s) were implemented to target improvements in patient QOL. Similarly, evidence is limited to indicate if any single transitional care intervention component is effective in improving QOL for patients with acute conditions. Additional research is needed to either power studies to test individual components or examine if specific mechanisms of action are effective in improving QOL for patients with acute conditions.

Reporting of facilitator training was limited in the studies included in this systematic review; training and characteristics of the training process are known to influence intervention outcomes [47], and future studies should ensure reporting of this critical component in order to support optimal outcomes with translation of evidence to practice. In addition, half of studies in this review did not describe or make available the intervention materials provided to or used with study participants. Studies that discussed intervention materials had vague descriptions and companion articles to provide more details were not identified by authors. Describing study materials in detail and making materials available when disseminating findings can increase the likelihood of intervention adaptation or replication [48]. Researchers should consider adopting the Template for Intervention Description and Replication (TIDieR) checklist and guide for better transparency in reporting [49].

Culture, race and ethnicity are often under-addressed in intervention research [50]. Although culture informs human behavior [50, 51], there has been limited incorporation of culture into research, particularly into development of evidence-based interventions [52]. None of the studies in this present review described cultural tailoring of their intervention. In addition, although race and ethnicity has been shown to influence response to intervention, few intervention studies report study findings by race or ethnicity to provide additional information on the efficacy or effectiveness of the intervention for various races [53, 54]. Studies outside the United States did not report demographic differences by race or ethnicity, and it is unclear if any were present; no studies in this review described findings by racial/ethnic groups. As incorporating culture, race, and ethnicity into all phases of the research process can help to reduce health disparities [55], future scholars should report the racial and ethnic composition of their sample, work to identify if needs differ by race and ethnicity in order to develop culturally-sensitive interventions, and consider analyzing findings by these important subgroups.

More specifically, researchers should design studies that allow space and time for cultural tailoring and should refer to existing resources for guidance on cultural tailoring of interventions, such as the NIH cultural framework for health and other publications on this topic [50, 56–58]. Interventions can be culturally tailored to address needs and preferences of racial/ethnic minorities in multiple ways, often starting by gathering information on or from relevant stakeholders from the cultural or racial/ethnic groups of focus, by conducting literature reviews on cultural differences, or by conducting qualitative and/or quantitative studies to assess the group’s needs and preferences [57]. Next, researchers should integrate the input from their stakeholders into the preliminary intervention protocol and materials and also consider translating intervention materials to language(s) used by the relevant stakeholders, where necessary [57]. Subsequently, researchers should solicit additional qualitative feedback (often through individual or focus group interviews or use of usability testing strategies) from relevant stakeholders to determine their thoughts on the acceptability of the preliminary intervention protocol and materials; researchers should then use this feedback to refine the final intervention protocol and materials [57]. Finally, after conducting the full trial using the culturally tailored intervention protocol and materials, researchers should analyze findings to determine presence of differences by subgroups, and consider conducting qualitative interviews with participants from relevant cultural or racial/ethnic groups to determine if further changes are needed [57].

Findings from this present study can be used to inform development and testing of transitional care interventions in diverse patient populations. For instance, as no studies in this review focused on patients with traumatic brain injury, a patient population that has high rates of injury in younger adults [59], findings from this present study can be used to inform development and testing of transitional care interventions that aim to improve quality of life in younger adult TBI patients. In addition, more work is needed to determine which intervention components adequately address the needs of patients and families during the transition from hospital to home. Similarly, as the majority of intervention components described in this review focus on the patient, more work is needed to determine appropriate intervention components to address the needs of family members of patients with acute conditions. Finally, more research is needed to determine how to culturally tailor transitional care interventions to address the needs and preferences of patients with acute conditions of all races and ethnicities.

### Limitations

One limitation of this study includes risk of bias across studies due to variation in report of treatment intervention details and participant characteristics. Another limitation is that quantitative meta-analyses were not conducted because the interventions were too heterogeneous to be considered comparable and to draw a conclusion to recommend the benefit of any one approach. Other limitations are related to the nature of systematic literature reviews, including the risk of incomplete retrieval of identified research and reporting bias. To address risk of incomplete retrieval of identified research, our research team worked in pairs to conduct study selection and data extraction. To address reporting bias, we specify all data extraction items, and provide detailed overviews of data reported in our results.

## Conclusions

Findings suggest there is wide variation in the components incorporated into transitional care interventions designed to improve QOL of patients hospitalized with acute conditions, as well as QOL outcome measures used. Research is needed on the mechanisms of action used to improve QOL of patients transitioning home after an acute hospital stay. Future research should consider incorporating differences in culture, race and ethnicity into all phases of the research process in an effort to address and reduce any health disparities and should elaborate on the background and training of intervention facilitators and on study materials utilized.

## Supplementary Information

**Additional file 1:** Electronic search strategy and articles excluded during full-text review.

## Data Availability

Please contact the first author for underlying research materials.

## Abbreviations

QOL: Quality of life
PRISMA: Preferred Reporting Items for Systemic Review and Meta-Analysis
PROSPERO: International Prospective Register of Systematic Reviews
CINHAL: The Cumulative Index to Nursing and Allied Health Literature
SGRQ: Saint George's Respiratory Questionnaire
SF-36: Short-Form 36
MLHFQ: Minnesota Living with Heart Failure Questionnaire
NHP: Nottingham Health Profile
KDQOL-SFTM: Kidney Disease Quality of Life Short Form
FACT-B: Functional Assessment of Cancer Therapy-Breast
EQ-5D: EuroQOL-5D
TIDieR: Template for Intervention Description and Replication
