# Supplementary material for: Transitions of care interventions to improve quality of life among patients hospitalized with acute conditions: a systematic literature review
Source: Health Qual Life Outcomes. 2021 Jan 29;19:36. doi: 10.1186/s12955-021-01672-5 (PMC7845026; doi:10.1186/s12955-021-01672-5)
Supplement: Supplementary file 1 — Additional file 1: Electronic search strategy and articles excluded during full-text review. [file 12955_2021_1672_MOESM1_ESM.docx]

Appendix, File 1

| Electronic Search Strategy used in PubMed | | |
| --- | --- | --- |
| Set # |  | Results |
| 1  Transitional Care | "Transitional Care"[Mesh] OR "Patient Navigation"[Mesh] OR "Patient Care Management"[Mesh] OR "Continuity of Patient Care"[Mesh] OR "transitional care"[tiab] OR "transition care"[tiab] OR "care transition"[tiab] OR "care transitions"[tiab] OR "patient navigation"[tiab] OR "case management"[tiab] OR "healthcare navigation"[tiab] OR "health care navigation"[tiab] OR "health system navigation"[tiab] OR "care coordination"[tiab] OR "coordinating care"[tiab] OR "care coordinators"[tiab] OR "discharge plan"[tiab] OR "discharge planning"[tiab] OR ((transition[tiab] OR transitional[tiab]) AND care[tiab]) | 929,402 |
| 2  Hospitals | "Hospitals"[Mesh] OR "Hospitalization"[Mesh] OR "Inpatients"[Mesh] OR hospital[tiab] OR hospitals[tiab] OR hospitalization[tiab] OR hospitalized[tiab] OR inpatient[tiab] OR inpatients[tiab] | 1,411,439 |
| 3  Outpatient | "Outpatients"[Mesh] OR home[tiab] OR homes[tiab] OR "home-based"[tiab] OR community[tiab] OR communities[tiab] OR outpatients[tiab] OR outpatient[tiab] | 882,215 |
| 4  Quality of Life | "Quality of Life"[MeSH Terms] OR "quality of life"[tiab] OR "life quality"[tiab] OR QoL[tiab] OR HRQOL[tiab] OR "satisfaction with life"[tiab] OR "life satisfaction"[tiab] | 321,016 |
| 5 | 1 AND 2 AND 3 AND 4 | 3,381 |
| 6 | 5 NOT (animals[mh] NOT humans[mh]) | 3,379 |
| 7 | 6 NOT (Editorial[ptyp] OR Letter[ptyp] OR Case Reports[ptyp] OR Comment[ptyp]) | 3,295 |

Note: Mesh = Medical Subject Headings; tiab = title/abstract; mh = MeSH Headings; ptyp = publication type

Appendix, File 2

**Articles Excluded During Full-Text Review**

1. Aboumatar, H., Naqibuddin, M., Chung, S., Chaudhry, H., Kim, S.W., Saunders, J., Bone, L., Gurses, A.P., Knowlton, A., Pronovost, P. and Putcha, N. (2018). Effect of a program combining transitional care and long-term self-management support on outcomes of hospitalized patients with chronic obstructive pulmonary disease: A randomized clinical trial. JAMA,*320*(22), 2335–2343. doi:10.1001/jama.2018.17933
2. Albert N. M. (2016). A systematic review of transitional-care strategies to reduce rehospitalization in patients with heart failure. *Heart & Lung: The Journal of Critical Care*, *45*(2), 100–113. <https://doi.org/10.1016/j.hrtlng.2015.12.001>
3. Allen, K., Hazelett, S., Jarjoura, D., Hua, K., Wright, K., Weinhardt, J., & Kropp, D. (2009). A randomized trial testing the superiority of a postdischarge care management model for stroke survivors. *Journal of Stroke and Cerebrovascular Diseases*, *18*(6), 443–452. <https://doi.org/10.1016/j.jstrokecerebrovasdis.2009.02.002>
4. Balinsky, W., & Muennig, P. (2003). The costs and outcomes of multifaceted interventions designed to improve the care of congestive heart failure in the inpatient setting: A review of the literature. *Medical Care Research and Review*, *60*(3), 275–293. <https://doi.org/10.1177/1077558703254697>
5. Bates, J., Sharratt, M., & King, J. (2014). Successful outsourcing: Improving quality of life through integrated support services. *Healthcare Management Forum*, *27*(1 Suppl), S58–S78. <https://doi.org/10.1016/j.hcmf.2014.01.009>
6. Bell, K. R., Brockway, J. A., Hart, T., Whyte, J., Sherer, M., Fraser, R. T., Temkin, N. R., & Dikmen, S. S. (2011). Scheduled telephone intervention for traumatic brain injury: A multicenter randomized controlled trial. *Archives of Physical Medicine and Rehabilitation*, *92*(10), 1552–1560. <https://doi.org/10.1016/j.apmr.2011.05.018>
7. Beney, J., Devine, E. B., Chow, V., Ignoffo, R. J., Mitsunaga, L., Shahkarami, M., McMillan, A., & Bero, L. A. (2002). Effect of telephone follow-up on the physical well-being dimension of quality of life in patients with cancer. *Pharmacotherapy*, *22*(10), 1301–1311. <https://doi.org/10.1592/phco.22.15.1301.33480>
8. Berry, L. L., Rock, B. L., Smith Houskamp, B., Brueggeman, J., & Tucker, L. (2013). Care coordination for patients with complex health profiles in inpatient and outpatient settings. *Mayo Clinic Proceedings*, *88*(2), 184–194. <https://doi.org/10.1016/j.mayocp.2012.10.016>
9. Taylor, C., Harding, W. G., Bosworth, P. M., & Shropshire, D. (1970). Bridging the gap between hospital and community. *NLN Publications*, (20-1353), 13–26.
10. Bird, S., Noronha, M., & Sinnott, H. (2010). An integrated care facilitation model improves quality of life and reduces use of hospital resources by patients with chronic obstructive pulmonary disease and chronic heart failure. *Australian Journal of Primary Health*, *16*(4), 326–333. <https://doi.org/10.1071/PY10007>
11. Black, S., Frazier, S., Haydo, M., A nurse practitioner directed, interdisciplinary model for heart failure transitional care. *PCNA Annual Symposium Winning Abstracts*, 424-425.
12. Carroll, A., & Dowling, M. (2007). Discharge planning: communication, education and patient participation. *British Journal of Nursing*, *16*(14), 882–886. <https://doi.org/10.12968/bjon.2007.16.14.24328>
13. Chow, Susan. (2006). The effects of a nurse-led case management programme on patients undergoing peritoneal dialysis: A randomized controlled trial.
14. Clark, A., & Nadash, P. (2004). The effectiveness of a nurse-led transitional care model for patients with congestive heart failure. *Home Healthcare Nurse*, *22*(3), 160–162. <https://doi.org/10.1097/00004045-200403000-00005>
15. Coleman, E. A., Rosenbek, S. A., & Roman, S. P. (2013). Disseminating evidence-based care into practice. *Population Health Management*, *16*(4), 227–234. <https://doi.org/10.1089/pop.2012.0069>
16. Cui, Y., Moriyama, M., Chayama, K., Liu, Y., Ya, C., Muzembo, B. A., & Rahman, M. M. (2019). Efficacy of a self-management program in patients with chronic viral hepatitis in China. *BMC Nursing*, *18*, 44. <https://doi.org/10.1186/s12912-019-0366-7>
17. Cyrille, N. B., & Patel, S. R. (2017). Late in-hospital management of patients hospitalized with acute heart failure. *Progress In Cardiovascular Diseases*, *60*(2), 198–204. <https://doi.org/10.1016/j.pcad.2017.05.003>
18. Dawes, H. A., Docherty, T., Traynor, I., Gilmore, D. H., Jardine, A. G., & Knill-Jones, R. (2007). Specialist nurse supported discharge in gynaecology: A randomised comparison and economic evaluation. *European Journal of Obstetrics, Gynecology, and Reproductive Biology*, *130*(2), 262–270. <https://doi.org/10.1016/j.ejogrb.2006.02.002>
19. Fens, M., Vluggen, T., van Haastregt, J. C., Verbunt, J. A., Beusmans, G. H., & van Heugten, C. M. (2013). Multidisciplinary care for stroke patients living in the community: A systematic review. *Journal of rehabilitation medicine*, *45*(4), 321–330. <https://doi.org/10.2340/16501977-1128>
20. Hekmatpou, D., Mohammad Baghban, E., & Mardanian Dehkordi, L. (2019). The effect of patient care education on burden of care and the quality of life of caregivers of stroke patients. *Journal of Multidisciplinary Healthcare*, *12*, 211–217. <https://doi.org/10.2147/JMDH.S196903>
21. Iseler, J. I., Wierenga, K. L., Shaid, E. C., & Hirschman, K. (2019). Implications of transitional care interventions on hospital readmissions in patients with destination therapy left ventricular assist devices. *Research and Theory for Nursing Practice*, *33*(1), 81–96. <https://doi.org/10.1891/1541-6577.33.1.81>
22. Kimman, M. L., Dirksen, C. D., Voogd, A. C., Falger, P., Gijsen, B. C., Thuring, M., Lenssen, A., van der Ent, F., Verkeyn, J., Haekens, C., Hupperets, P., Nuytinck, J. K., van Riet, Y., Brenninkmeijer, S. J., Scheijmans, L. J., Kessels, A., Lambin, P., & Boersma, L. J. (2011). Nurse-led telephone follow-up and an educational group programme after breast cancer treatment: Results of a 2 × 2 randomised controlled trial. *European Journal of Cancer*, *47*(7), 1027–1036. <https://doi.org/10.1016/j.ejca.2010.12.003>
23. Le Berre, M., Maimon, G., Sourial, N., Guériton, M., & Vedel, I. (2017). Impact of transitional care services for chronically ill older patients: A systematic evidence review. *Journal of the American Geriatrics Society*, *65*(7), 1597–1608. <https://doi.org/10.1111/jgs.14828>
24. Mistiaen, P., Francke, A. L., & Poot, E. (2007). Interventions aimed at reducing problems in adult patients discharged from hospital to home: A systematic meta-review. *BMC Health Services Research, 7(*1), 47.
25. Nagae, H., Tanigaki, S., Okada, M., Katayama, Y., Norikoshi, C., Nishina, Y., & Sakai, M. (2013). Identifying structure and aspects that 'continuing nursing care' used in discharge support from hospital to home care in Japan. *International Journal of Nursing Practice*, *19 Suppl 2*, 50–58. <https://doi.org/10.1111/ijn.12044>
26. Nowak, K., Nagle, L., Bernardo, A. (1998). Review: Case management programmes improve patient outcomes. *Evidence-Based Nursing,1*,128. <http://dx.doi.org/10.1136/ebn.1.4.128>
27. Oh, E. G., Kim, J. H., & Lee, H. J. (2019). Effects of a safe transition programme for discharged patients with high unmet needs. *Journal of Clinical Nursing*, *28*(11-12), 2319–2328. <https://doi.org/10.1111/jocn.14831>
28. Olson, D. M., Bettger, J. P., Alexander, K. P., Kendrick, A. S., Irvine, J. R., Wing, L., ... & Graffagnino, C. (2011). Transition of care for acute stroke and myocardial infarction patients: From hospitalization to rehabilitation, recovery, and secondary prevention. In *Database of Abstracts of Reviews of Effects (DARE): Quality-assessed Reviews*. Centre for Reviews and Dissemination (UK).
29. Perez, A., Garcia, N., de Juan, L., Grocin, I., Igarzabal, A., & Alkiza, M.E. (2018). Impact on the use of services and quality of life of a continuity of care unit for multi-pathological patients. *International Journal of Integrated Care, 18(s2), 148.* <http://doi.org/10.5334/ijic.s2148>
30. Plant, N. A., Kelly, P. J., Leeder, S. R., D'Souza, M., Mallitt, K. A., Usherwood, T., Jan, S., Boyages, S. C., Essue, B. M., McNab, J., & Gillespie, J. A. (2015). Coordinated care versus standard care in hospital admissions of people with chronic illness: A randomised controlled trial. *The Medical Journal of Australia*, *203*(1), 33–38. <https://doi.org/10.5694/mja14.01049>
31. Preen, D. B., Bailey, B. E., Wright, A., Kendall, P., Phillips, M., Hung, J., Hendriks, R., Mather, A., & Williams, E. (2005). Effects of a multidisciplinary, post-discharge continuance of care intervention on quality of life, discharge satisfaction, and hospital length of stay: A randomized controlled trial. *International Journal for Quality in Health care*, *17*(1), 43–51. <https://doi.org/10.1093/intqhc/mzi002>
32. Pugh, L. C., Tringali, R. A., Boehmer, J., Blaha, C., Kruger, N. R., Capauna, T. A., Bryan, Y., Robinson, J., Belmont, D., Young, M., & Xie, S. (1999). Partners in care: A model of collaboration. *Holistic Nursing Practice*, *13*(2), 61–65. https://doi.org/10.1097/00004650-199901000-00010
33. Reed, P. H., & Hulton, L. J. (2017). Community health workers in collaboration with case managers to improve quality of life for patients with heart failure. *Professional Case Management*, *22*(3), 144–148. <https://doi.org/10.1097/NCM.0000000000000222>
34. Reed, P.H. (2016). Using community health workers in collaboration with nurse case managers in effecting change in quality of life for heart failure patients.
35. Rezapour-Nasrabad, R. (2018). Transitional care model: Managing the experience of hospital at home. *Electronic Journal of General Medicine, 15*(5), em73. <https://doi.org/10.29333/ejgm/93445>
36. Richards, S., & Coast, J. (2003). Interventions to improve access to health and social care after discharge from hospital: A systematic review. *Journal of Health Services Research & Policy*, *8*(3), 171–179. <https://doi.org/10.1258/135581903322029539>
37. Shahsavari, H., Zarei, M., & Aliheydari Mamaghani, J. (2019). Transitional care: Concept analysis using Rodgers' evolutionary approach. *International Journal of Nursing Studies*, *99*, 103387. <https://doi.org/10.1016/j.ijnurstu.2019.103387>
38. Stamp, K. D., Machado, M. A., & Allen, N. A. (2014). Transitional care programs improve outcomes for heart failure patients: An integrative review. *The Journal of Cardiovascular Nursing*, *29*(2), 140–154. <https://doi.org/10.1097/JCN.0b013e31827db560>
39. Stevenson D. (2004). Training informal caregivers of patients with stroke improved patient and caregiver quality of life and reduced costs. *Evidence-Based Nursing*, *7*(4), 118. <https://doi.org/10.1136/ebn.7.4.118>
40. Tho, P. C., & Ang, E. (2016). The effectiveness of patient navigation programs for adult cancer patients undergoing treatment: A systematic review. *JBI database of Systematic Reviews and Implementation Reports*, *14*(2), 295–321. <https://doi.org/10.11124/jbisrir-2016-2324>
41. Turner, B. J., Fleming, J. M., Ownsworth, T. L., & Cornwell, P. L. (2008). The transition from hospital to home for individuals with acquired brain injury: A literature review and research recommendations. *Disability and Rehabilitation*, *30*(16), 1153–1176. <https://doi.org/10.1080/09638280701532854>
42. Van Spall, H., Rahman, T., Mytton, O., Ramasundarahettige, C., Ibrahim, Q., Kabali, C., Coppens, M., Brian Haynes, R., & Connolly, S. (2017). Comparative effectiveness of transitional care services in patients discharged from the hospital with heart failure: A systematic review and network meta-analysis. *European Journal of Heart Failure*, *19*(11), 1427–1443. <https://doi.org/10.1002/ejhf.765>
43. Vloothuis, J. D., Mulder, M., Veerbeek, J. M., Konijnenbelt, M., Visser-Meily, J. M., Ket, J. C., Kwakkel, G., & van Wegen, E. E. (2016). Caregiver-mediated exercises for improving outcomes after stroke. *The Cochrane Database of Systematic Reviews*, *12*(12), CD011058. <https://doi.org/10.1002/14651858.CD011058.pub2>
44. Williams N. (2003). Nurse led transitional care improved health related quality of life and reduced emergency department use for heart failure. *Evidence-based Nursing*, *6*(1), 21. <https://doi.org/10.1136/ebn.6.1.21>
45. Zhang, P., Xing, F. M., Li, C. Z., Wang, F. L., & Zhang, X. L. (2018). Effects of a nurse-led transitional care programme on readmission, self-efficacy to implement health-promoting behaviours, functional status and life quality among Chinese patients with coronary artery disease: A randomised controlled trial. *Journal of Clinical Nursing*, *27*(5-6), 969–979. <https://doi.org/10.1111/jocn.14064>
46. (2015). Adjusting CM services in a transition-focused world. *Case Management Advisor.* 26(9): 103-104.
